# Supplementary material for: Tia1 dependent regulation of mRNA subcellular location and translation controls p53 expression in B cells
Source: Nat Commun. 2017 Sep 13;8:530. doi: 10.1038/s41467-017-00454-2 (PMC5597594; doi:10.1038/s41467-017-00454-2)
Supplement: Supplementary file 1 — Supplementary Information [file 41467_2017_454_MOESM1_ESM.pdf]

Description of Supplementary Files

File name: Supplementary Information
Description: Supplementary figures and supplementary table.

File name: Supplementary Data 1
Description: Annotated Tia1:RNA crosslink sites mapped to the genome in LPS- activated B cells. Two independent experiments shown.

File name: Supplementary Data 2
Description: Differential expression analysis of cellular mRNA abundance after DNA damage induction with etoposide.

File name: Supplementary Data 3
Description: Changes in ribosome-associated mRNA abundance after treatment with etoposide.

File name: Supplementary Data 4
Description: Changes in mRNA content associated to a given polysome fraction (changes in mRNA polysome distribution).

File name: Supplementary Data 5
Description: GO terms associated to differentially translated Tia1 mRNA targets in B cells (Gorilla and REVIGO analysis, related to figure 2H, iCLIP target definition - cut off = Minimum of 10 unique crosslinks in the 3'UTR).

File name: Supplementary Data 6
Description: Number and percentage of mRNA transcripts distributed in different polysome fractions.

File name: Peer review file

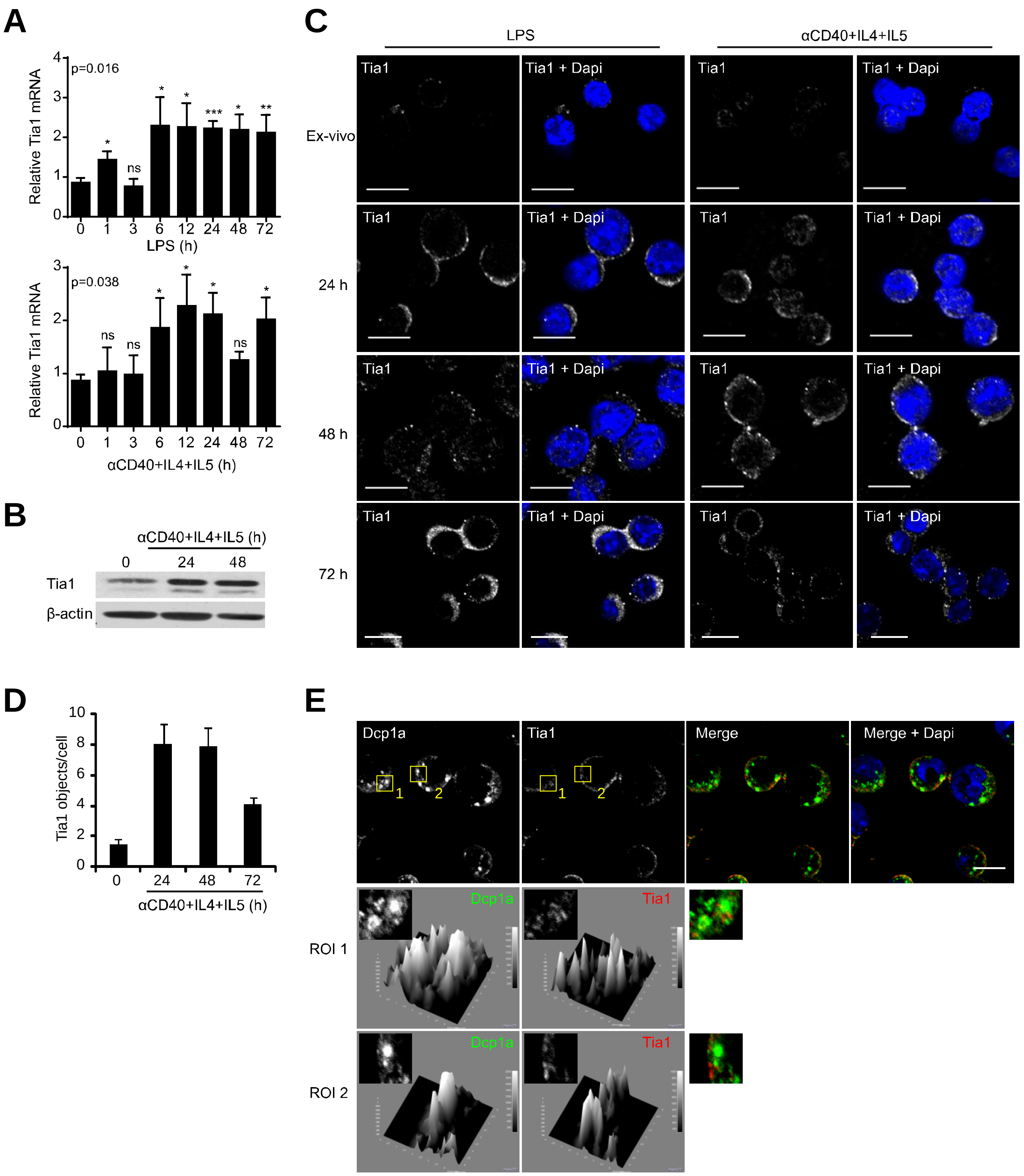
A
p=0.016
Relative Tia1 mRNA
LPS (h)
p=0.038
αCD40+IL4+IL5 (h)
B
αCD40+IL4+IL5 (h)
Tia1
β-actin
C
LPS
αCD40+IL4+IL5
Tia1
Tia1 + Dapi
Ex-vivo
24 h
48 h
72 h
D
Tia1 objects/cell
αCD40+IL4+IL5 (h)
E
Dcp1a
Tia1
Merge
Merge + Dapi
ROI 1
ROI 2

F

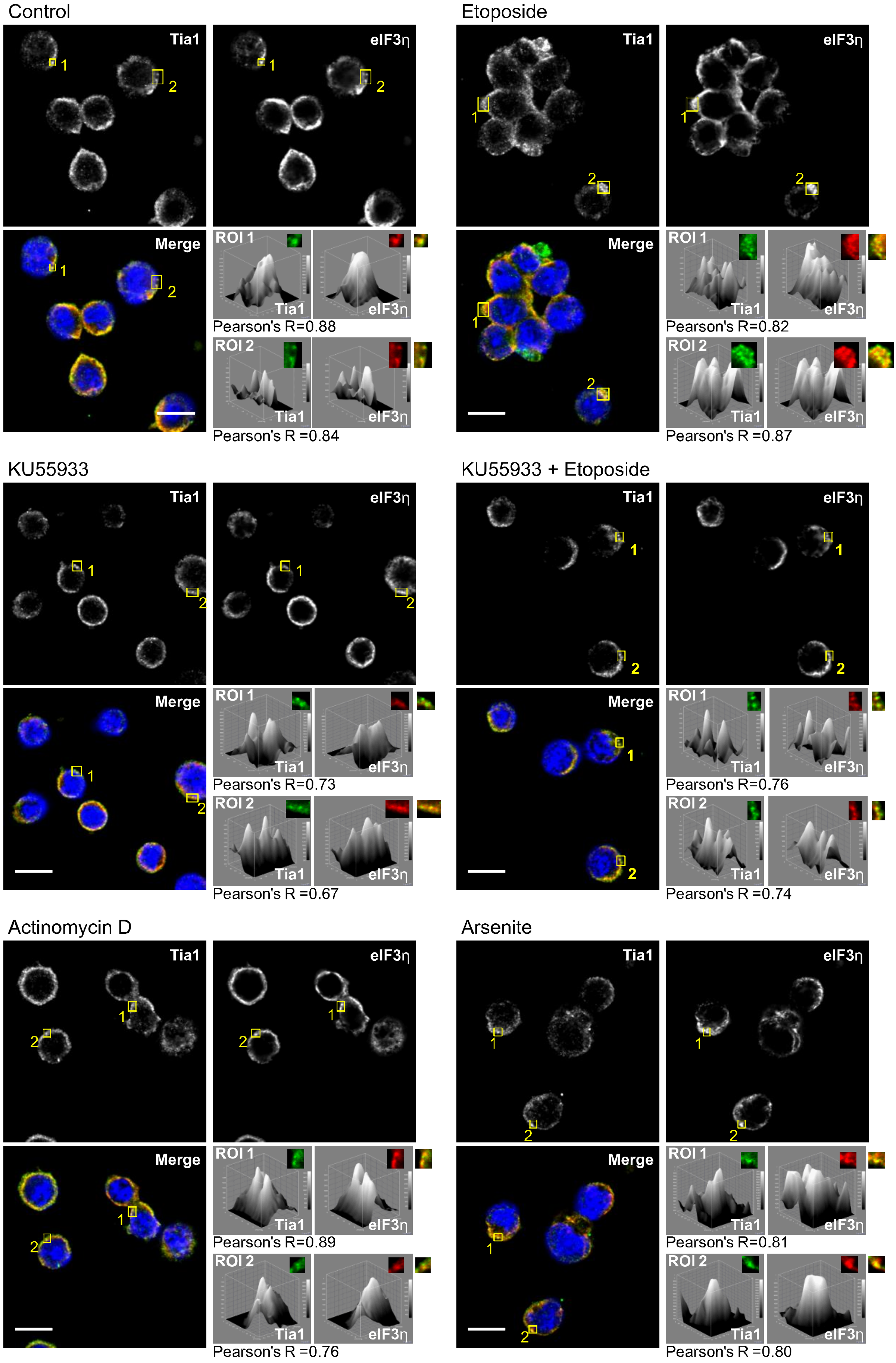
Control
Tia1
eIF3η
Merge
ROI 1
Pearson's R=0.88
ROI 2
Pearson's R =0.84
Etoposide
Tia1
eIF3η
Merge
ROI 1
Pearson's R=0.82
ROI 2
Pearson's R =0.87
KU55933
Tia1
eIF3η
Merge
ROI 1
Pearson's R=0.73
ROI 2
Pearson's R =0.67
KU55933 + Etoposide
Tia1
eIF3η
Merge
ROI 1
Pearson's R=0.76
ROI 2
Pearson's R =0.74
Actinomycin D
Tia1
eIF3η
Merge
ROI 1
Pearson's R=0.89
ROI 2
Pearson's R =0.76
Arsenite
Tia1
eIF3η
Merge
ROI 1
Pearson's R=0.81
ROI 2
Pearson's R =0.80

Supplementary fig. 1. Stress granules are formed upon B cell activation.

A, Time course analysis of Tia1 mRNA expression in B cells after activation with LPS or αCD40+IL4+IL5. Data from two independent experiments are shown as mean + s.e.m. (One-way analysis of variance (indicated p values) and unpaired t tests were performed comparing treated vs untreated samples, * $p<0.05$, ** $p<0.01$, *** $p<0.001$). **B,** Immunoblot analysis of Tia1 protein expression in B cells treated with αCD40+IL4+IL5. **C,** Confocal analysis of Tia1 subcellular location in B cells after activation with LPS or αCD40+IL4+IL5 for the indicated times. **D,** Number of Tia1-containing granules per cell in the confocal images shown in C. Quantitation was performed using Volocity Image software based on fluorescence accumulation within granules. A minimum of six confocal images from two independent experiments were analysed. Data shown as mean + s.d. **E,** Visualization of Dcp1a and Tia1 subcellular location in B cells activated with LPS for 48 hours. Dcp1a and Tia1 were detected using specific antibodies. Dapi was used for nucleus (DNA) staining. Bottom panels show a 3D fluorescence maps (Image J) of both Ddp1a and Tia1 fluorescence signal in two selected regions of interest (ROI). **F,** Confocal analysis of Tia1 and eIF3η in LPS-activated B cells that were treated or not with KU55933 (10 μM, 4h), etoposide (20 μM, 4 h), KU55933 plus etoposide (4 h), actinomycin D (5 μg/ml, 1h) or sodium arsenite (100 μM, 30 min). 3D fluorescence maps of both Tia1 and eIF3η were performed as in E. Pearson's correlation for Tia1 and eIF3η signal colocation is shown for both ROIs. A minimum of six confocal images from at least two independent experiments were analysed in C, E and F. (bar scale = 10 μm).

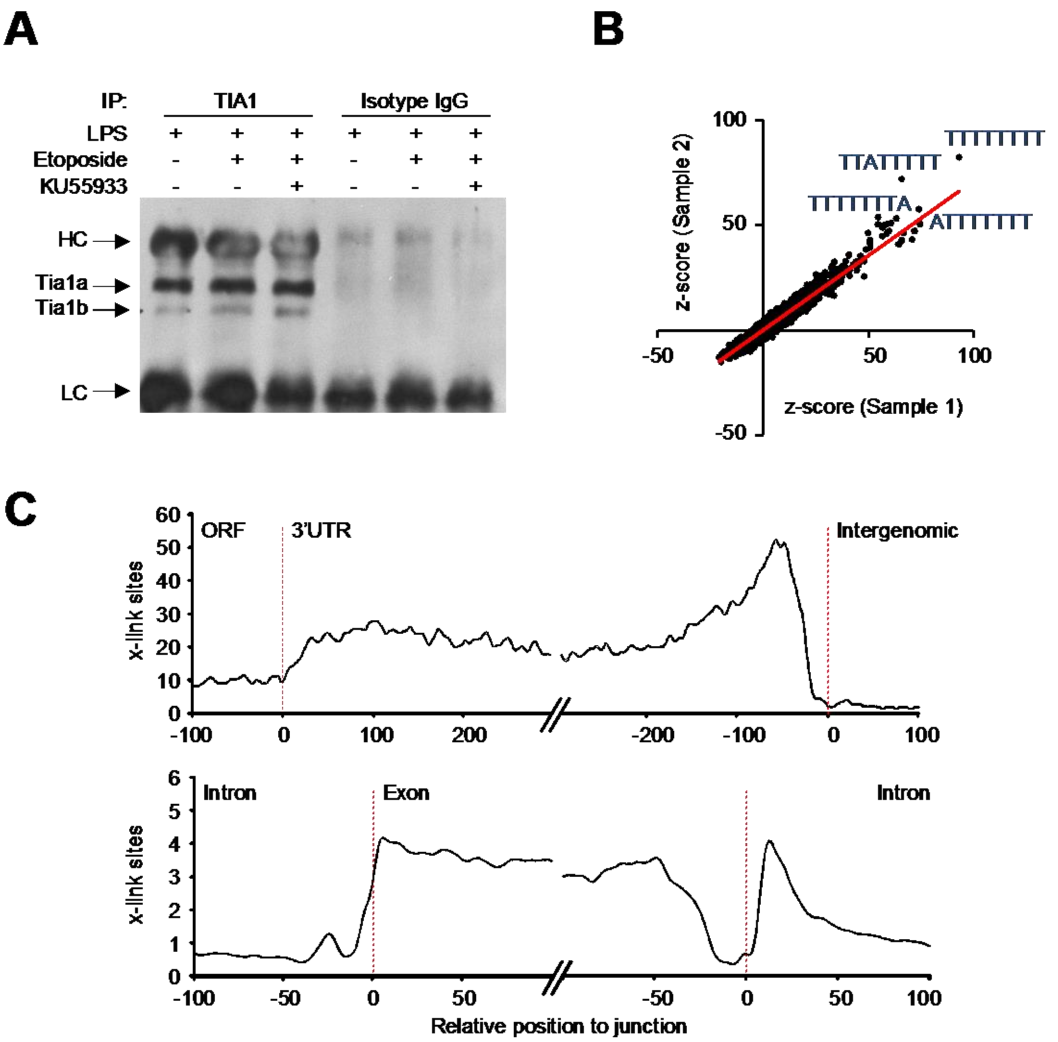

D

GO term	Description	frequency	semantic space	plot_size	log10 p-value
GO:0050789	regulation of biological process	46.58%	1.998	4.468	-14.7496
GO:0019222	regulation of metabolic process	25.26%	1.887	4.202	-9.0615
GO:0032502	developmental process	24.06%	-0.362	4.181	-7.2534
GO:0032879	regulation of localizat on	7.17%	-0.154	3.655	-6.6289
GO:0002682	regulation of immune system process	3.91%	4.467	3.392	-6.4271
GO:0023051	regulation of signaling	10.96%	1.98	3.839	-5.3645
GO:0002376	immune system process	8.70%	0.472	3.739	-5.0141
GO:0010646	regulation of cell communicat on	11.00%	1.111	3.841	-4.8447
GO:0050896	response to stimulus	36.21%	1.459	4.358	-4.6498
GO:0048583	regulation of response to st mulus	12.52%	7.056	3.897	-4.3716
GO:0030155	regulation of cell adhesion	1.35%	0.856	2.931	-4.3363
GO:0030334	regulation of cell migrat on	1.88%	-1.733	3.074	-4.0004
GO:0030029	actin fl ament-based process	2.69%	-0.257	3.229	-2.5986
GO:0070228	regulation of lymphocyte apoptot c process	0.23%	0.639	2.161	-2.58
GO:0040008	regulation of growth	2.44%	2.378	3.187	-2.5436
GO:0010608	posttranscript onal regulti on of gene expression	1.43%	-3.533	2.954	-2.3382
GO:0009605	response to external stimulus	7.34%	8.088	3.665	-2.0883
GO:0006109	regulation of carbohydrate metabolic process	0.54%	-4.014	2.537	-2.0223
GO:0019216	regulation of lipid metabolic process	0.94%	-4.916	2.772	-2.0223
GO:0034248	regulation of cellular amide metabolic process	0.07%	-5.159	1.633	-2.0039
GO:0001775	cell activat on	3.45%	-0.206	3.337	-1.7852
GO:0002634	regulation of germinal center format on	0.04%	6.625	1.362	-1.6904
GO:0006897	endocytosis	1.88%	-5.394	3.074	-1.6716
GO:0045730	respiratory burst	0.07%	-3.068	1.623	-1.6459

Supplementary fig. 2. Identification of Tia1 mRNA targets by iCLIP.

A, Immunoblot analysis of Tia1 immunoprecipitation using a Tia1- specific or an IgG isotype antibody. Cytoplasmic extract from LPS-activated B cells treated with Etoposide or Etoposide+KU55933 were used. **B,** Tia1 binding motif analysis shown as 8-mers enriched in the two independent iCLIP experiments performed. **C,** RNA map showing Tia1 binding enrichment associated to the ORF-3'UTR and 3'UTR-intergenomic boundaries (top panel), or the intron-exon and exon-intron boundaries (bottom panel). Quantification x-link sites was normalised by the genomic segment size. **D,** Table of GO terms enriched in the list of Tia1 mRNA targets in B cells (GOrilla and REViGO analysis, related to figure 1F).

A

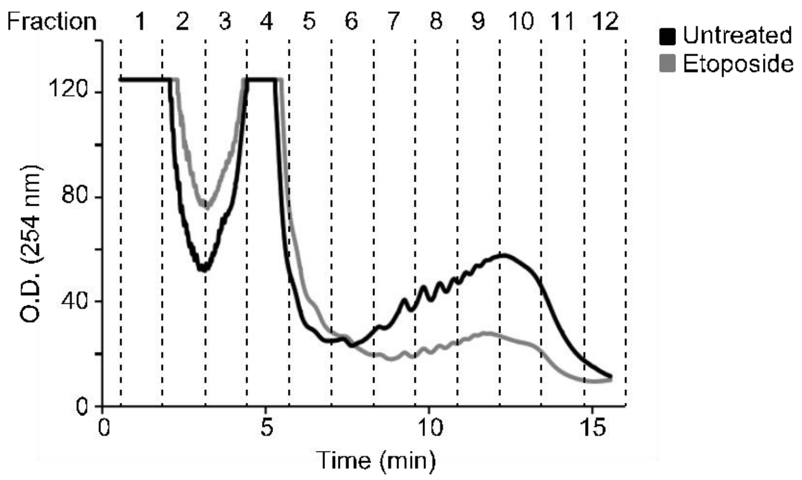

B

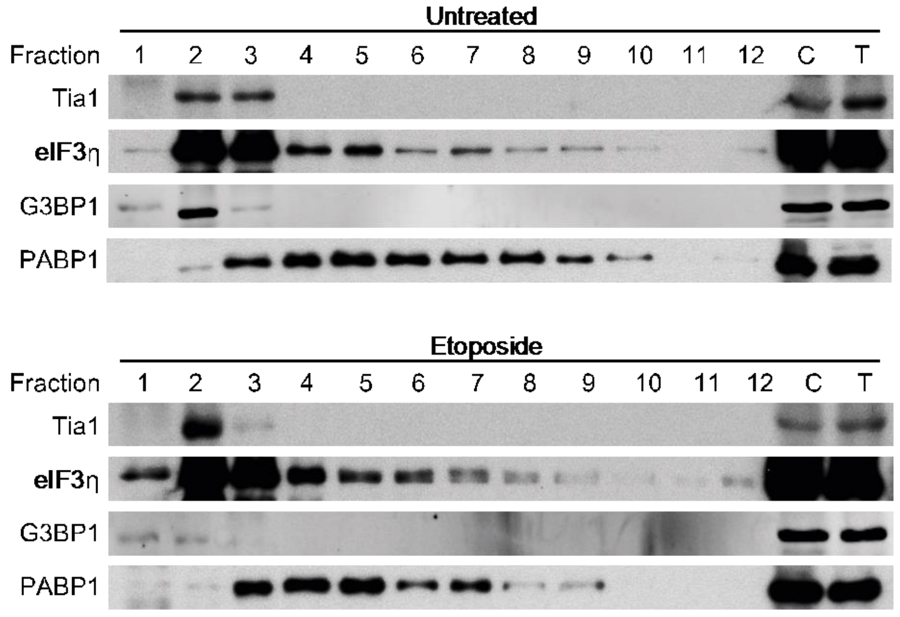

Supplementary fig. 3. Etoposide alters mRNA distribution in polysomes in activated B cells.

A, Polysome profiles of LPS- activated B cells treated or not with etoposide (20 μM) for 4 hours. Data is representative of 6 independent experiments. **B,** Immunoblot analysis of Tia1, eIF3η, G3BP1 and PABP1 in the different polysome fractions (1.4 ml fractions as indicated in A), in cytoplasmic protein extracts (C) and total cell lysates (T) from LPS-activated B cells treated or not with etoposide for 4 hours. Data are from one of the two independent experiments performed.

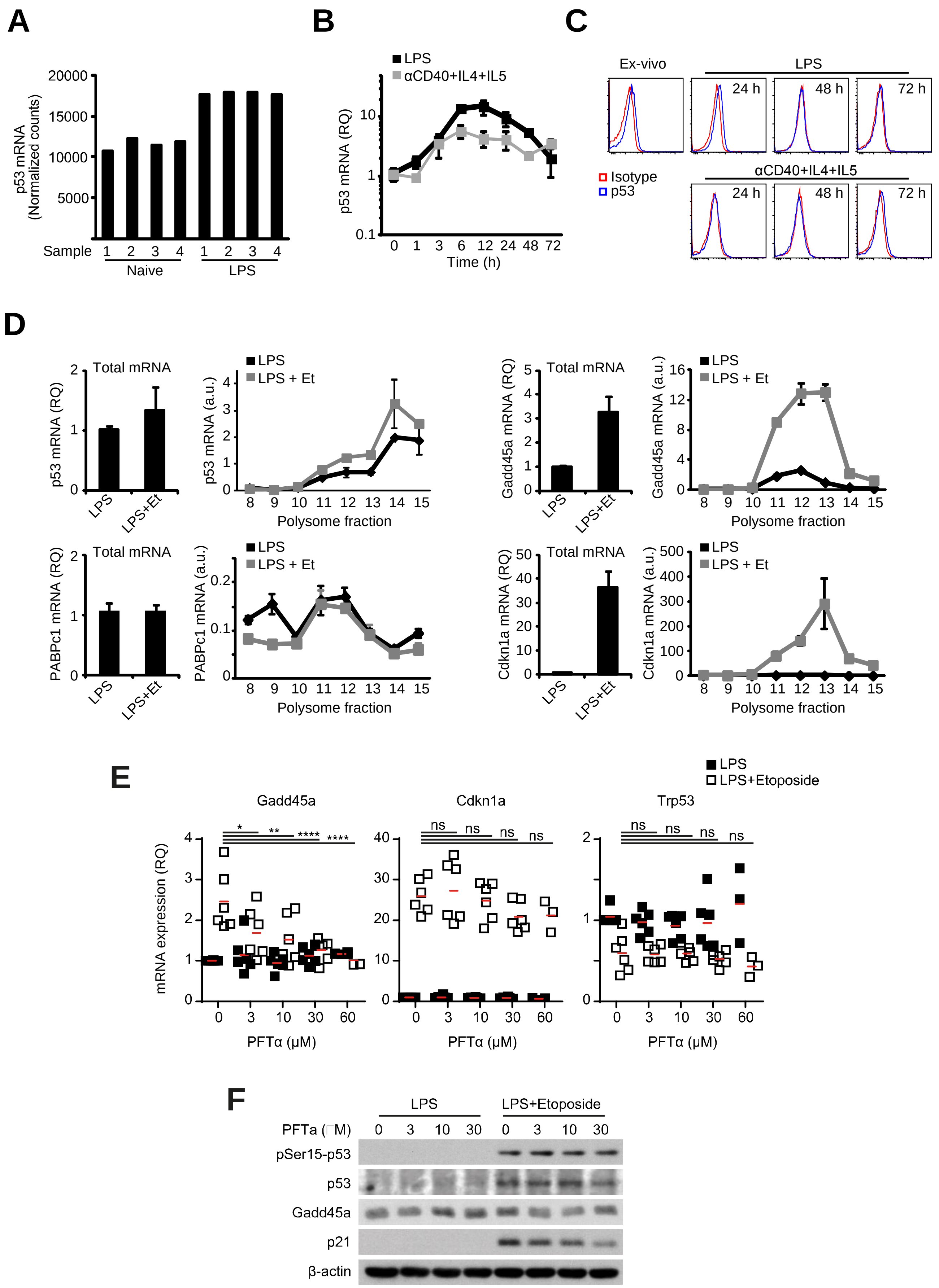
A
p53 mRNA (Normalized counts)
Sample
Naive
LPS
B
LPS
αCD40+IL4+IL5
p53 mRNA (RQ)
Time (h)
C
Ex-vivo
LPS
24 h
48 h
72 h
Isotype
p53
αCD40+IL4+IL5
D
Total mRNA
p53 mRNA (RQ)
p53 mRNA (a.u.)
LPS + Et
Polysome fraction
Gadd45a mRNA (RQ)
Gadd45a mRNA (a.u.)
PABPc1 mRNA (RQ)
PABPc1 mRNA (a.u.)
Cdkn1a mRNA (RQ)
Cdkn1a mRNA (a.u.)
E
LPS+Etoposide
Gadd45a
Cdkn1a
Trp53
mRNA expression (RQ)
PFTα (µM)
F
PFTa (μM)
pSer15-p53
p53
Gadd45a
p21
β-actin

Supplementary fig. 4. p53 mRNA translation increases without changes in mRNA abundance upon induction of DNA damage.

A, p53 mRNA expression in naïve and LPS-activated B cells. Data from mRNAseq libraries (GSE62129) is shown as DESeq2 normalised counts (each of the 4 biological replicates is identified by the number). **B,** RT-qPCR time course analysis of p53 mRNA expression in B cells activated with LPS or αCD40+IL4+IL5. Data from 2 independent experiments. Data shown as mean + s.d. **C,** p53 protein expression in B cells treated with LPS or αCD40+IL4+IL5 measured by intracellular flow analysis. **D,** RT-qPCR analysis of total mRNA levels and mRNA polysome distribution of p53, Gadd45a, PABPc1 and Cdkn1a in LPS- activated B cells treated or not with etoposide. Total mRNA levels were normalised by Hprt mRNA abundance and are shown as a relative quantification (RQ) of the LPS-treated B cell control samples. mRNA polysome distribution is shown arbitrary units (a.u.). Data from three biological replicates are shown as mean + s.d. **E,** Analysis by RT-qPCR of Gadd45a, Cdkn1a and p53 mRNA expression in LPS-activated B cells treated with the indicated doses of Pifithrin-α (PFTα) 1 hour prior induction of DNA damage with etoposide. Data from 6 biological replicates collected in two independent experiments are shown with the exception of cells treated with 60 μM of PFTα for which data from three biological replicates from one experiment are shown. Two-way ANOVA and Bonferroni post-test analysis was performed (ns= non-significant, *$p<0.05$, **$p<0.01$, ***$p<0.001$, ****$p<0.0001$). **F,** Immunoblot analysis of Gadd45a, Cdkn1a, pSer15-p53, p53 and β-actin in total protein extracts of B cells treated like in E. Data are from one of the two independent experiments performed.

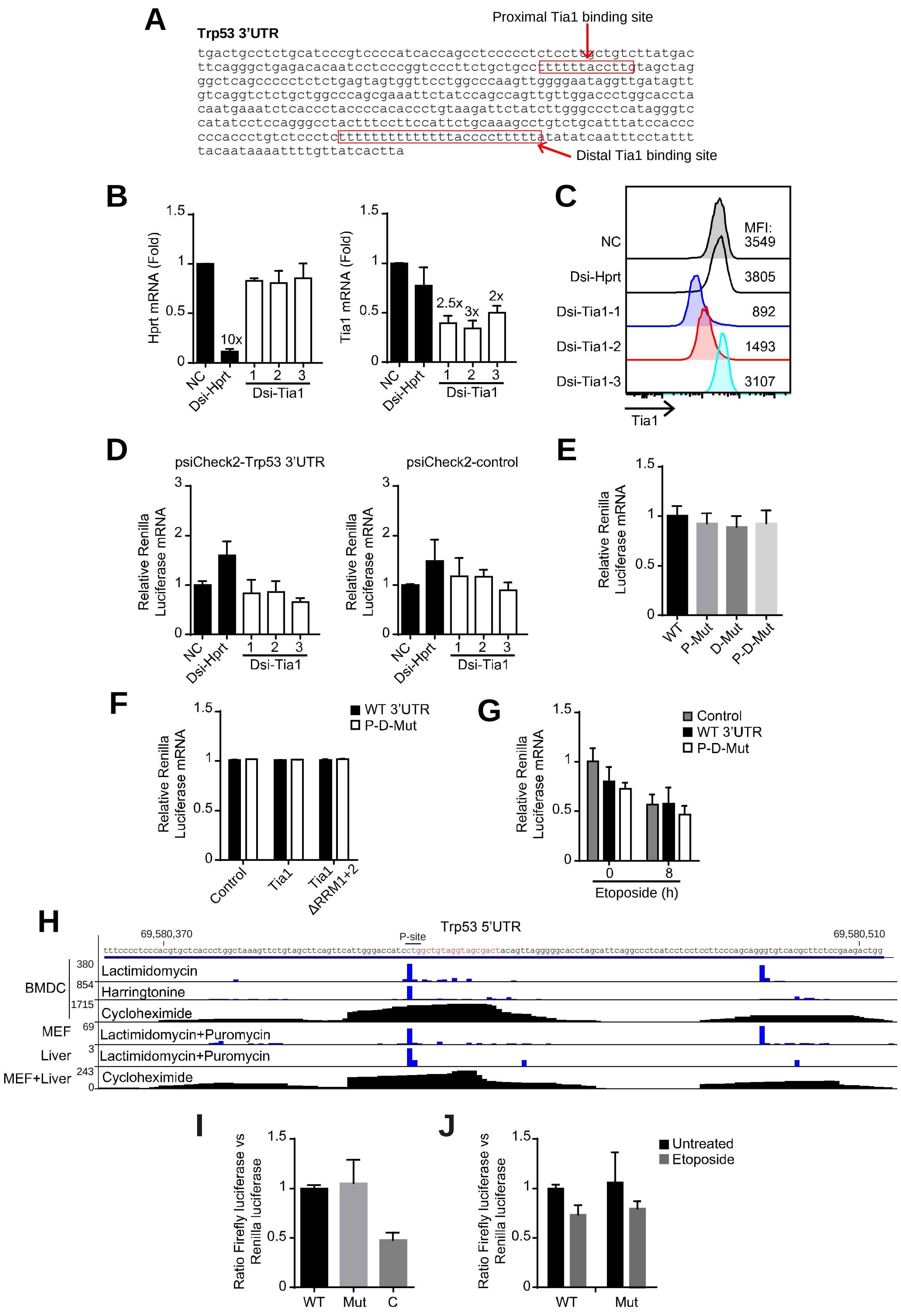

A
Trp53 3'UTR
Proximal Tia1 binding site
tgactgcctctgcatcccgtccccatcaccagcctccccctctccttgctgtcttatgac
ttcagggctgagacacaatcctcccggtcccttctgctgcctttttaccttgtagctag
ggctcagccccctctctgagtagtggttcctggcccaagttggggaataggttgatagtt
gtcaggtctctgctggcccagcgaaattctatccagccagttgttggaccctggcaccta
caatgaaatctcaccctaccccacaccctgtaagattctatcttgggccctcatagggtc
catatcctccagggcctactttccttccattctgcaaagcctgtctgcatttatccaccc
cccaccctgtctccctctttttttttttttttaccccttttttatatatcaatttcctattt
tacaataaaatttgttatcactta
Distal Tia1 binding site
B
Hprt mRNA (Fold)
1.5
1
0.5
0
10x
NC
Dsi-Hprt
1
2
3
Dsi-Tia1
Tia1 mRNA (Fold)
2.5x
3x
2x
C
NC
Dsi-Hprt
Dsi-Tia1-1
Dsi-Tia1-2
Dsi-Tia1-3
MFI:
3549
3805
892
1493
3107
Tia1
D
psiCheck2-Trp53 3'UTR
psiCheck2-control
Relative Renilla Luciferase mRNA
3
2
1
0
E
WT
P-Mut
D-Mut
P-D-Mut
F
WT 3'UTR
P-D-Mut
Control
Tia1
Tia1 ΔRRM1+2
G
Control
WT 3'UTR
P-D-Mut
0
8
Etoposide (h)
H
Trp53 5'UTR
69,580,370
P-site
69,580,510
tttcccctcccacgtgctcaccctggctaaagttctgtagcttcagttcattgggaccatcctggctgtaggtagcgactacagttagggggcacctagcattcaggccctcatcctccttcccagcagggtgtcacgcttctccgaagactgg
BMDC
380
Lactimidomycin
854
Harringtonine
1715
Cycloheximide
MEF
69
Lactimidomycin+Puromycin
Liver
3
Lactimidomycin+Puromycin
MEF+Liver
243
Cycloheximide
0
I
Ratio Firefly luciferase vs Renilla luciferase
WT
Mut
C
J
Untreated
Etoposide
WT
Mut

Supplementary fig. 5. Tia1 deficiency does not alter p53 mRNA abundance.

A, Murine p53 3'UTR nucleotide sequence cloned to generate the psiCheck2-p53 3'UTR (WT) plasmid construct. Red boxes highlight the proximal and the distal sequences bound by Tia1. **B,** Quantitation by qPCR of Tia1 and Hprt mRNA abundance in HEK293 cells co-transfected with a psiCheck2 control or a psiCheck2-p53 3'UTR (WT) plasmid construct and a gene-specific dsi-RNA or a scramble dsi-RNA (negative control, NC). The fold reduction in expression compared to the negative control is indicated. Data from three independent experiments is shown. **C,** Representative flow cytometry histograms showing Tia1 protein expression in HEK293 cells transfected with Tia1 dsi-RNAs, Hprt dsiRNA or scramble dsi-RNA. Data from one of the two independent experiments is shown (MFI=Median fluorescence intensity). **D,** Quantitation by qPCR of renilla luciferase mRNA in HEK293 cells described in B. Data was normalised by firefly luciferase mRNA expression to control for differences in transfection efficiency and is shown relative to the negative control (NC). **E,** Relative quantitation by qPCR of renilla luciferase mRNA in HEK293 cells transfected with a psiCkeck2- p53 3'UTR (WT), a psiCkeck2- p53 3'UTR P-mutant (P-Mut), a psiCkeck2- p53 3'UTR D-mutant (D-Mut) or a psiCkeck2- p53 3'UTR P-D-mutant (P-D-Mut) plasmid construct. Data was normalised by firefly luciferase mRNA expression and is shown as relative to the expression in cells transfected with the psiCkeck2- p53 3'UTR (WT) plasmid. **F,** mRNA abundance of renilla luciferase in HEK293 cells co-transfected with a psiCheck2-p53 3'UTR (WT) or a psiCkeck2- p53 3'UTR P-D-mutant (P-D-Mut) plasmid and a MIGR1-Tia1, a MIGR1-Tia1 ΔRRM1+2 or a MIGR1-empty control plasmid. Data was normalised by firefly luciferase mRNA expression and is shown as relative to the control. **G,** Analysis by qPCR of renilla luciferase mRNA expression in HEK293 transfected with a psiCheck2 control, a psiCheck2-p53 3'UTR (WT) or a psiCkeck2- p53 3'UTR P-D-mutant (P-D-Mut) plasmid construct and treated after 24 hours with etoposide (20 μM) for 8 hours. Data was normalised by firefly luciferase mRNA expression and is shown as relative to the expression in untreated HEK293 cells transfected with a psiCheck2 control vector. **H,** Visualization of ribosome footprints mapped to p53 mRNA using GWIPS-viz. Data from bone marrow-derived dendritic cells (BMDC) treated with the translation initiation inhibitors lactimidomycin or harringtonine, and from mouse embryonic fibroblasts (MEFs) and liver cells treated with lactimidomycin plus puromycin, was used to identify the p53 IRES sequence occupying the ribosome p-site. Ribo-seq coverage tracks from BMDC and MEF+Liver samples treated with cycloheximide are shown. **I,** Relative quantitation by qPCR of wild type (WT) and IRES-mutated (mut) bicistronic Trp53 IRES reporter mRNAs in transfected HEK293 cells. The ratio between two qPCR assays detecting firefly luciferase CDS and renilla luciferase CDS is shown. The ratio in control HEK293 cells transfected with a psiCheck2 empty vector was also quantified (C). **J,** Analysis of wild type (WT) and IRES-mutated (mut) bicistronic Trp53 IRES reporter mRNAs in HEK293 cells treated or not with etoposide (20 μM) for 8 hours. Data shown as mean+s.d. are from three independent experiments in B, D, E, F, G, I and J.

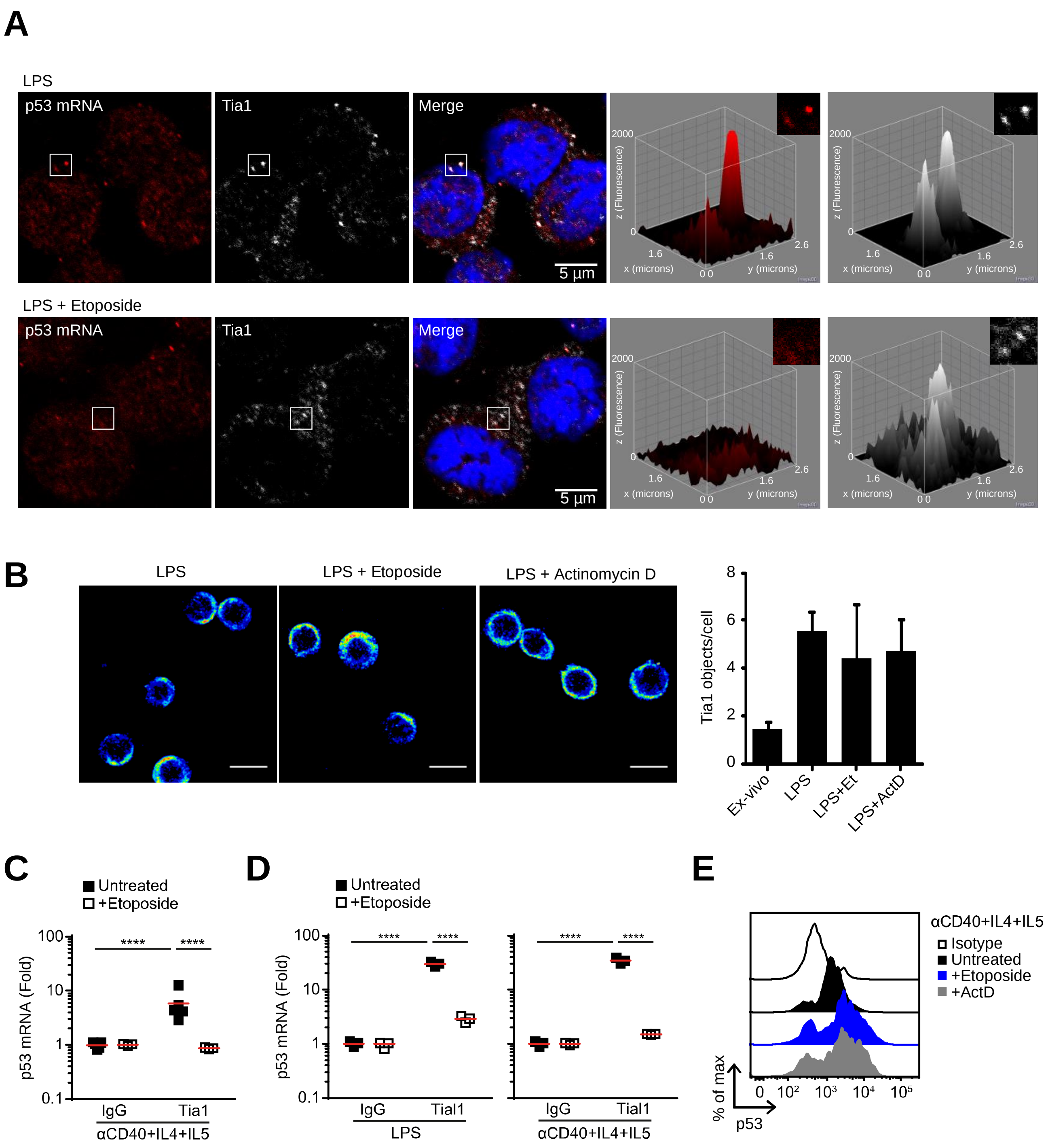

Supplementary fig. 6. Etoposide induces p53 mRNA release from stress granules.

A, (relates to figure 3H) Confocal analysis of p53 mRNA and Tia1 protein in LPS-activated B cells treated or not with etoposide. Fluorescence intensity 3D maps from a selected region (white boxes) were generated to visualise Tia1 protein and p53 mRNA accumulation in cytoplasmic stress granules. Z-axis of 3D maps relates to fluorescence intensity (FI). Representative images are from one of the two independent experiments performed. A minimum of five confocal images were analysed in each experiment. **B,** Confocal imaging of Tia1- containing granules in activated B cells treated with etoposide or actinomycin D (ActD). Left panels, representative confocal images showing Tia1 fluorescent signal as a spectrum of colours (from black to red).

Red colour marks fluorescence signal saturation associated to Tia1 containing granules. Right panel, quantification of Tia1- containing granules per cell. Data is shown as the mean +SEM of values from a minimum of six confocal images from at least two independent experiments. **C,** Tia1 protein:p53 mRNA co-immunoprecipitation in B cells activated with αCD40+IL4+IL5 and treated or not with etoposide. Data from two independent experiments (1-3 experimental replicates in each) are shown. Mann-Whitney test was performed for statistical analysis of the data (****p<0.0001). **D,** Co-immunoprecipitation of Tial1 protein and p53 mRNA in B cells activated either with LPS (left) or αCD40+IL4+IL5 (right). Etoposide was used to induce DNA damage. An IgG isotype antibody was used as negative control. Data from three biological replicates collected in one independent experiment are shown. Mann-Whitney test was performed for statistical analysis of the data (****p<0.0001). **E,** Mouse p53 3'UTR sequence cloned into psiCheck2-p53 3'UTR Full. Nucleotide sequence removed in the psiCheck2-p53 3'UTR Short vector is underlined. **F,** Intracellular flow cytometry of p53 protein expression in B cells activated with αCD40+IL4+IL5 for 48 hour prior treatment with etoposide or actinomycin D.

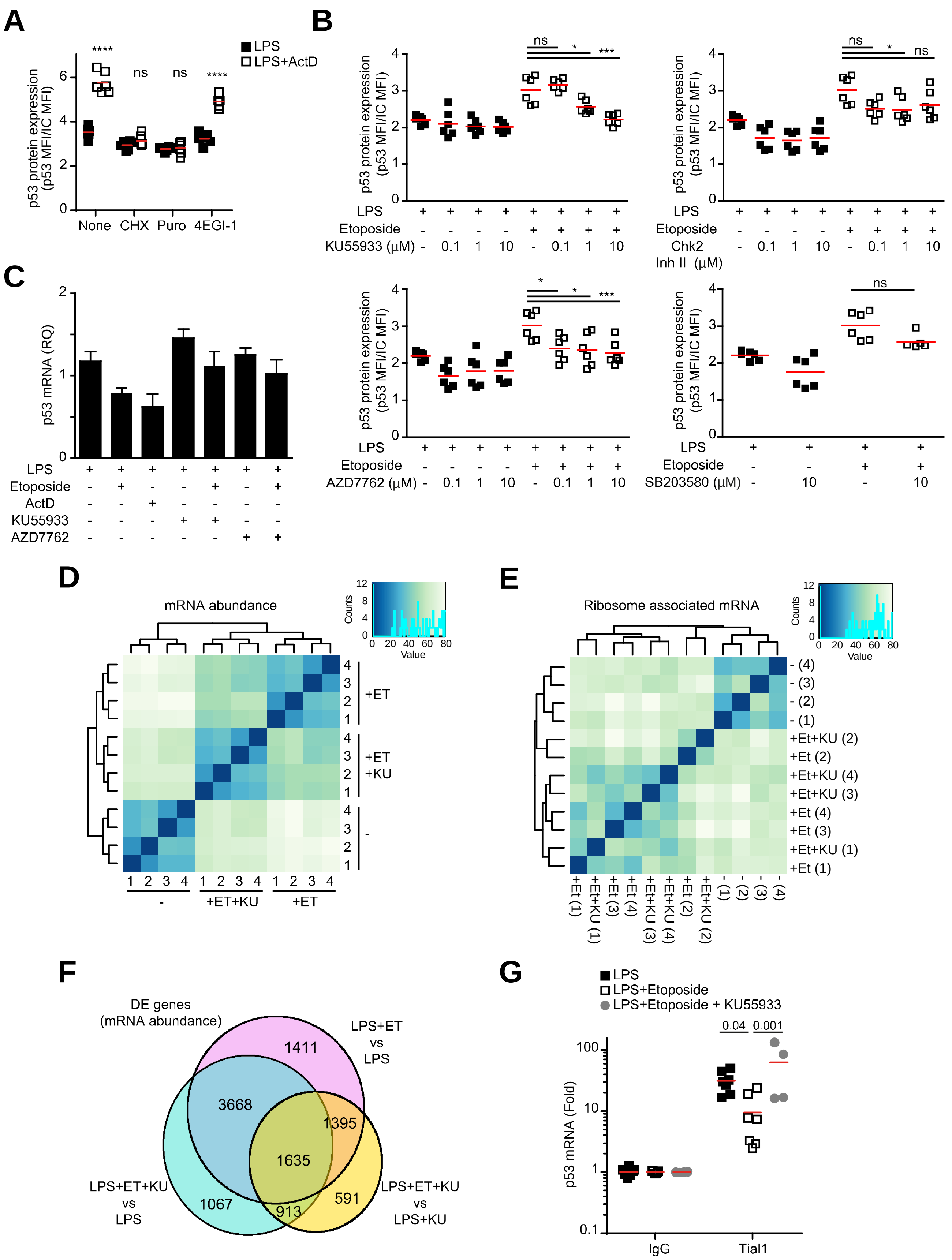

A
B
C
D
E
F
G
LPS
LPS+ActD
p53 protein expression (p53 MFI/IC MFI)
None CHX Puro 4EGI-1
Etoposide
KU55933 (μM)
Chk2 Inh II (μM)
AZD7762 (μM)
SB203580 (μM)
p53 mRNA (RQ)
ActD
KU55933
AZD7762
mRNA abundance
Ribosome associated mRNA
Counts
Value
+ET
+ET +KU
+ET+KU
DE genes (mRNA abundance)
LPS+ET vs LPS
LPS+ET+KU vs LPS
LPS+ET+KU vs LPS+KU
1411
3668
1395
1635
1067
913
591
LPS+Etoposide
LPS+Etoposide + KU55933
p53 mRNA (Fold)
IgG
Tial1
0.04
0.001

Supplementary fig. 7. ATM kinase modulates p53 mRNA translation.

A, Analysis by flow cytometry of p53 protein expression in LPS-activated B cells treated with the inhibitors of translation cycloheximide, puromycin or 4EGI-1 prior induction of DNA damage with actinomycin D. Data are from six biological replicates collected in two independent experiments. Two-way ANOVA and Bonferroni post-test analysis was performed (ns= non-significant, ****$p<0.0001$). **B,** Quantitation of p53 protein levels in B cells incubated with the ATM inhibitor KU55933, the Chk1/2 inhibitor AZD7762, the Chk2 inhibitor II or the p38 inhibitor SB203580 prior induction of DNA damage with etoposide (related to Figure 4A). p53 expression was quantified by intracellular flow cytometry in A and B. Data are shown as the median of fluorescence intensity (MFI) of p53 normalized by the MFI of an isotype antibody control. Data are from six biological replicates from the two independent experiments performed. (Mann-Whitney test, ns= non-significant, * $p<0.05$, ***$p<0.001$). **C,** p53 mRNA expression in activated B cells incubated with KU55933 or AZD7762 prior treatment with etoposide. Actinomycin D (Act D) was alternative used to induce DNA damage. Data are shown as the mean value from three biological replicates + s.d. **D,** Cluster analysis of the transcriptome of B cells treated with LPS (-), LPS+Etoposide (+ Et) or LPS+Etoposide+KU55933 (+Et +KU). B cell transcriptome was measured by mRNAseq (each number identifies one of 4 biological replicates). **E,** Cluster analysis of the ribosome- associated mRNA translatome measured by Polysome fractionation followed by 3'end sequencing (PolyRibo-3' RNA-seq) (each of the 4 biological replicates is identified by a number). **F,** Venn- diagram of differentially expressed mRNA (total mRNA abundance) on the pairwise comparisons of the conditions described in D. **G,** Tial1 protein: p53 mRNA co-immunoprecipitation in activated B cells treated with etoposide in the presence of the ATM inhibitor KU55933. An IgG isotype antibody was used as negative control. Data from the three independent experiments performed are shown as relative enrichment compared to the LPS-treated IgG control.

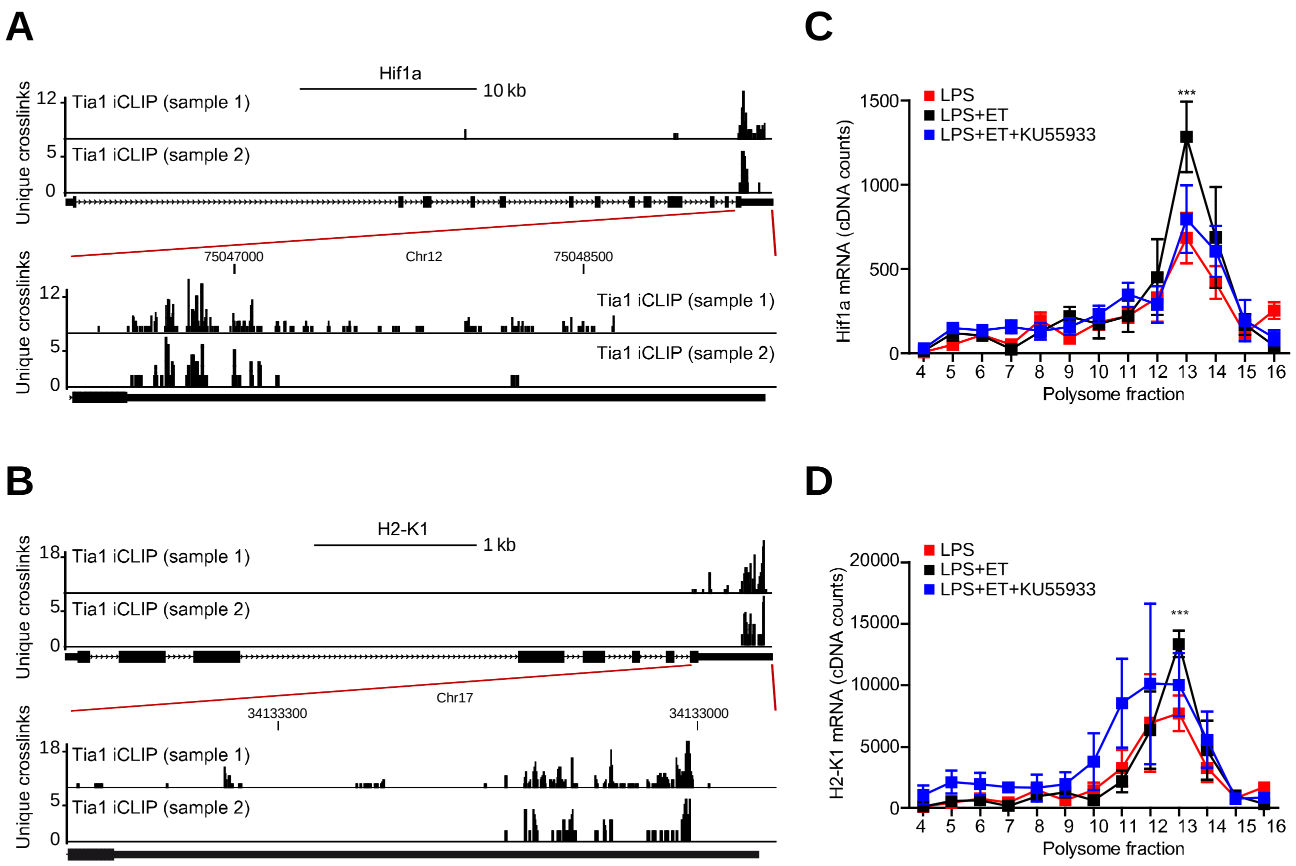

Supplementary fig. 8. Tia1 binds to Hif1α and H2-K1 3'UTRs.

A, Map of Tia1- binding sites across Hif1α gene locus. **B,** Polysome profile of Hif1α quantified by PolyRibo-3' RNA-seq. **C,** Tia1- binding sites mapped to H2-K1 gene locus. Tia1 binding to the last exon was magnified in A and C (bottom genomic tracks). Data from the two iCLIP experiments performed are shown. **D,** Polysome profile of Hif1α from PolyRibo-3' RNA-seq experiments. LPS-activated B cells were treated or not with the ATM inhibitor KU55933 prior induction of DNA damage with etoposide. Data shown as mean cDNA counts + s.e.m. (n=4, post-hoc pairwise t-test, *** p<0.001 for both comparisons: LPS vs LPS+etoposide (LPS+ET) and LPS+Etoposide vs LPS+Etoposide+KU55933 (LPS+ET+KU55933)).

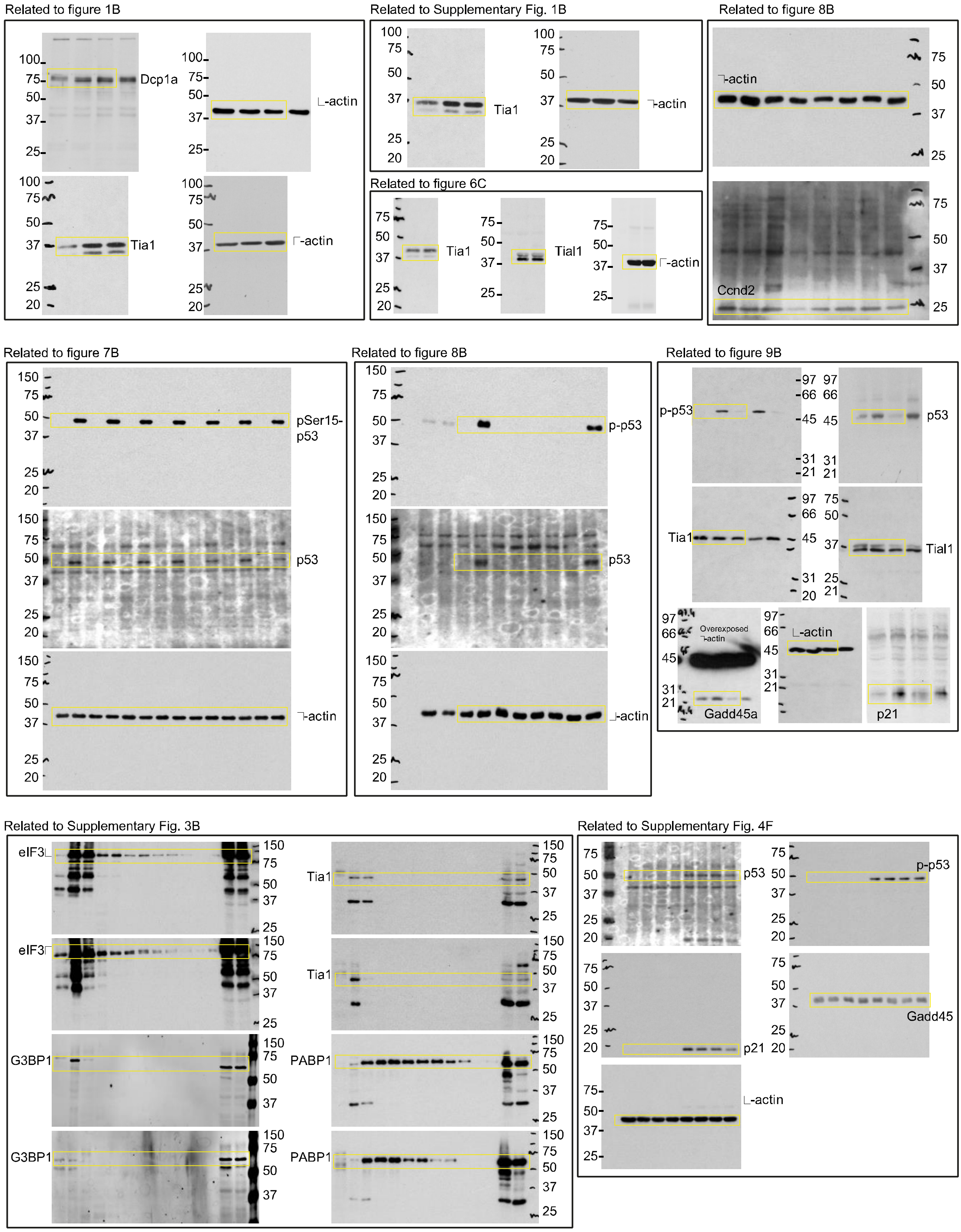

Supplementary fig. 9. Immunoblots.

Supplementary Table 1. Reagents, antibodies, qPCR primers and probes.

Reagent and antibodies	Source	Cat. Number	Application
LPS (*E.coli 0127:B8*)	Sigma Aldrich	L3129	In vitro cell activation
Interleukin-4 (IL4)	Peprotech	214-14	In vitro cell activation
Interleukin-5 (IL5)	Sigma Aldrich	I1145	In vitro cell activation
anti-CD40 antibody (clone 3/23 clone)	In home purified	-	In vitro cell activation
B Cell Isolation Kit, mouse	Milteny Biotec	130-090-862	B cell isolation
RPMI 1640 Medium (Dutch modification)	ThermoFisher Scientific	22409-015	Cell culture
2-mercaptoethanol	ThermoFisher Scientific	31350010	Cell culture
L-glutamine	ThermoFisher Scientific	25030081	Cell culture
Penicillin-Streptomycin	ThermoFisher Scientific	15140122	Cell culture
Fetal calf serum	ThermoFisher Scientific	10270-106	Cell culture
Etoposide	Sigma Aldrich	E1383	Cell culture
Cycloheximide	Sigma Aldrich	C7698	Cell culture/Polysome fractionation
Dimethyl sulfoxide	Sigma Aldrich	D2650	Cell culture
KU55933	Tocris	3544	Cell culture
SB203580	Cell Signalling Technologies	5633	Cell culture
Chk2 inhibitor II	Sigma Aldrich	C3742	Cell culture
AZD7762	Axon MedChem	1399	Cell culture
Actinomycin D	Sigma Aldrich	A9415	Cell culture
MG132	Sigma Aldrich	M8699	Cell culture
Sodium arsenite	Sigma Aldrich	35000-1L-R	Cell culture
Puromycin	Sigma Aldrich	P8833	Cell culture
SJ172550	Sigma Aldrich	S7451	Cell culture
HBX41108	Tocris	4285	Cell culture
P005091	Tocris	4733	Cell culture
Lactacystin	Calbiochem	2267	Cell culture
Nutlin-3	Calbiochem	444143	Cell culture
Pifithrin-alpha	Calbiochem	506132	Cell culture
p53 Activator III RITA	Calbiochem	506149	Cell culture
4EGI-1	Calbiochem	324517	Cell culture
p53 antibody (clone G59-12) coupled to PE	BD Biosciences	557027 - set	Flow cytometry
Mouse IgG1 isotype control coupled to PE	BD Biosciences	557027 - set	Flow cytometry
BD Cytofix/Cytoperm™ solution	BD Biosciences	554722	Flow cytometry
BD Perm/Wash buffer	BD Biosciences	554723	Flow cytometry
Pierce™ BCA Protein Assay Kit	ThermoFisher Scientific	23225	Protein quantitation
Poly-L-lysine solution	Sigma Aldrich	P8920	IF
Goat anti-Tia1 (C-20) IgG antibody	Santa Cruz Technologies	sc-1751	WB/IF/iCLIP
Rabbit anti-Tia1 IgG antibody	Sigma Aldrich	SAB2102429	WB/IF
Goat anti-Tial1 (C-18) IgG antibody	Santa Cruz Technologies	sc-1749	WB
Rabbit anti-Tia1 monoclonal IgG antibody Alexa Fluor 647	Abcam	EPR9304	Flow cytometry
Rabbit anti-GFP polyclonal IgG antibody Alexa Fluor 488	ThermoFisher Scientific	A21311	Flow cytometry
Mouse anti-HuR monoclonal (3A2) antibody	BD Biosciences	566341	Flow cytometry
Rabbit anti-Dcp1a rabbit serum	Dr. Jens Lykke-Anderson	-	WB/IF
Goat anti-eIF3eta (N-20) IgG antibody	Santa Cruz Technologies	sc-16377	IF
Goat anti-rabbit IgG (H+L) secondary antibody Alexa Fluor 488	ThermoFisher Scientific	A11034	IF
Goat anti-rabbit IgG1 secondary antibody Alexa Fluor 488	ThermoFisher Scientific	A21121	IF
Goat anti-rabbit IgG (H+L) secondary antibody Alexa Fluor 555	ThermoFisher Scientific	A21428	IF
Donkey anti-goat IgG (H+L) secondary antibody Alexa Fluor 647	ThermoFisher Scientific	A21447	IF
Sheep anti-digoxigenin antibody	ThermoFisher Scientific	PA1-85378	RNA FISH
Rabbit polyclonal anti-Gadd45a antibody	Santa Cruz Technologies	sc-797	WB
Goat polyclonal anti-p53 antibody	Santa Cruz Technologies	sc1315	WB
Mouse monoclonal anti-p53 (Pab 246) antibody	Santa Cruz Technologies	sc-100	WB

Rabbit anti-p53 (Ser15) antibody	Cell Signalling Technologies	9284L	WB
Mouse monoclonal anti-p21 antibody	BD Biosciences	556431	WB
Rabbit polyclonal anti-p21 antibody	Santa Cruz Technologies	sc-397	WB
Rabbit polyclonal anti-PABP1 antibody	Cell Signalling Technologies	4992	WB
Mouse monoclonal anti-G3BP1 antibody	Santa Cruz Technologies	sc-81940	WB
Goat IgG isotype control	Santa Cruz Technologies	sc-3887	iCLIP
Mouse monoclonal anti-actin b antibody	Sigma Aldrich	A1978	WB
TRIzol® Reagent	ThermoFisher Scientific	15596-026	RNA extraction
Platinum® SYBR® Green qPCR SuperMix-UDG w/ROX	ThermoFisher Scientific	11744500	qPCR
Platinum® Quantitative PCR SuperMix-UDG	ThermoFisher Scientific	11730025	qPCR
TruSeq Stranded mRNA Sample Prep Kit	Illumina	RS-122-2103	mRNAseq
RNase I (Cloned) 100 U/µl	ThermoFisher Scientific	AM2294	iCLIP
Dynabeads® Protein G for Immunoprecipitation	ThermoFisher Scientific	10004D	iCLIP
T4 Polynucleotide Kinase	NEB	M0201S	iCLIP
Fermentas* Shrimp Alkaline Phosphatase (SAP)	ThermoFisher Scientific	EF0511	iCLIP
T4 RNA Ligase 1 (ssRNA Ligase)	NEB	M0204S	iCLIP
Proteinase K, recombinant, PCR Grade	Roche	311887	iCLIP
Acid-Phenol:Chloroform, pH 4.5 (with IAA, 125:24:1)	ThermoFisher Scientific	AM9722	iCLIP
SuperScript® III Reverse Transcriptase	ThermoFisher Scientific	18080-093	iCLIP/RNA FISH
CircLigase™ II ssDNA Ligase	Epicentre/Illumina	CL9021K	iCLIP
Fermentas* FastDigest* BamHI	Fermentas	FD0054	iCLIP
Phusion® Hot Start Flex 2X Master Mix	NEB	F-548S	iCLIP
Dual-Luciferase® Reporter Assay System	Promega	E1910	Luciferase assay
MAXIscript SP6/T7 Transcription Kit	ThermoFisher Scientific	AM1320	RNA FISH
PfuUltra II Fusion HS DNA Polymerase	Agilent Genomics	600670	RNA FISH
T7 RNA Polymerase	Promega	P2075	RNA FISH/PolyRiboSeq
Digoxigenin-11-2'-deoxy-uridine-5'-triphosphate, alkali-stable	Roche	11093088910	RNA FISH
RNase H	ThermoFisher Scientific	EN0201	RNA FISH
QIAquick Nucleotide Removal Kit	Qiagen	28304	RNA FISH
Ribonucleoside Vanadyl Complex	NEB	S1402S	RNA FISH
Donkey serum	Sigma Aldrich	D9663	IF
RNaseOUT™ Recombinant Ribonuclease Inhibitor	ThermoFisher Scientific	10777019	RNA FISH/iCLIP/qPCR/Polysome fractionation/RIP
XhoI	NEB	R0146S	Cloning
NotI	NEB	R0189S	Cloning
BglII	NEB	R0144S	Cloning
SalI	NEB	R3138S	Cloning
EcoRI	NEB	R3101S	Cloning
TOPO TA Cloning Kit with PCR2.1 TOPO	ThermoFisher Scientific	K4500-01	Cloning
psiCHECK™-2 Vector	Promega	C8021	Cloning
LipoFectamine 2000	ThermoFisher Scientific	11668027	Luciferase assay
Lipofectamine® RNAiMAX Reagent	ThermoFisher Scientific	13778075	DsiRNA and luciferase assay
Sucrose	Sigma Aldrich	84097	Polysome fractionation
32P-g-ATP 3000Ci(111TBq)/mmol (250 uCi=9.25MBq)	Perkin Elmer	NEG002A	iCLIP
Protease inhibitors	Sigma Aldrich	P8340	WB/iCLIP/RIP
Dynabeads® Protein G	ThermoFisher Scientific	10004D	iCLIP/RIP
Pierce™ Protein A/G Magnetic Beads	ThermoFisher Scientific	88802	RIP
QIAquick PCR Purification Kit	Qiagen	28104	PolyRiboSeq
Turbo DNase I, RNase-free	ThermoFisher Scientific	AM1907	iCLIP/RIP/PolyRiboSeq/qPCR
MessageAmp™ II aRNA Amplification Kit	ThermoFisher Scientific	AM1751	PolyRiboSeq
RNase A, DNase and protease-free	ThermoFisher Scientific	EN0531	PolyRiboSeq
TruSeq Stranded mRNA Library Prep Kit	Illumina	RS-122-2103	RNAseq
TriFECTa Kit DsiRNA duplex - hs.Ri.TIA1.13.1	Integrated DNA Technologies (IDT)	156347972	DsiRNA and luciferase assay
TriFECTa Kit DsiRNA duplex - hs.Ri.TIA1.13.2	Integrated DNA Technologies (IDT)	156347969	DsiRNA and luciferase assay
TriFECTa Kit DsiRNA duplex - hs.Ri.TIA1.13.3	Integrated DNA Technologies (IDT)	156347966	DsiRNA and luciferase assay

HPRT-S1 Positive Control DsiRNA	Integrated DNA Technologies (IDT)	51-01-08-02	DsiRNA and luciferase assay
Negative Control DsiRNA	Integrated DNA Technologies (IDT)	51-01-14-03	DsiRNA and luciferase assay
**Taqman assays, primers and oligonucleotides**	**Source**	**Cat. Number**	**Sequence**
Dcp1a Taqman assay	ThermoFisher Scientific	Mm00460131_m1	-
Dcp2 Taqman assay	ThermoFisher Scientific	Mm01264052_m1	-
Edc4 Taqman assay	ThermoFisher Scientific	Mm00725090_m1	-
Tnrc4a Taqman assay	ThermoFisher Scientific	Mm00523487_m1	-
Xrn1 Taqman assay	ThermoFisher Scientific	Mm00496326_m1	-
Cnot8 Taqman assay	ThermoFisher Scientific	Mm00518365_m1	-
Tia1 Taqman assay	ThermoFisher Scientific	Mm00441748_m1	-
Tial1 Taqman assay	ThermoFisher Scientific	Mm00437049_m1	-
Elavl1 Taqman assay	ThermoFisher Scientific	Mm00516011_m1	-
Cdkn1a Taqman assay	ThermoFisher Scientific	Mm01303209_m1	-
Eukaryotic 18S rRNA Taqman assay	ThermoFisher Scientific	Hs99999901_s1	-
beta-2 microglobulin Taqman assay	ThermoFisher Scientific	Mm00437762_m1	
Mouse Elavl1 (Forw)	Sigma Aldrich	-	GGATGACATTGGGAGAACGAAT
Mouse Elavl1 (Rev)	Sigma Aldrich	-	TGTCCTGCTACTTTATCCCGAA
18S rRNA (Forw)	Sigma Aldrich	RT-qPCR	GAGGTAGTGACGAAAAATAACAAT
18S rRNA (Rev)	Sigma Aldrich		TTGCCCTCCAATGGATCCT
Mouse PABPc1 (Forw)	Sigma Aldrich	RT-qPCR	CTCAACTAAGACCAAGTCCTCGCT
Mouse PABPc1 (Rev)	Sigma Aldrich		CATGACTCGTGGAACCTGTGAG
Mouse Gadd45a (Forw)	Sigma Aldrich	RT-qPCR	GCTGCTGCTACTGGAGAACGA
Mouse Gadd45a (Rev)	Sigma Aldrich		GATCCTTCCATTGTGATGAATGTG
Mouse Trp53 (Forw)	Sigma Aldrich	RT-qPCR	CGACCTATCCTTACCATCATCACA
Mouse Trp53 (Rev)	Sigma Aldrich		TTCTGTACGGCGGTCTCTCC
Renilla luciferase (Forw)	Sigma Aldrich	RT-qPCR	TGCTGAACCTTCCAAAGAAAAT
Renilla luciferase (Rev)	Sigma Aldrich		CTTGGTGCTCGTAGGAGTAGTG
Firefly luciferase (Forw)	Sigma Aldrich	RT-qPCR	CCTGCTAACGACATTTACAACG
Firefly luciferase (Rev)	Sigma Aldrich		CTTAGACACGAACACCACGGTA
Human Tia1 (Forw)	Sigma Aldrich	RT-qPCR	GCCCAAGACTCTATACGTCGG
Human Tia1 (Rev)	Sigma Aldrich		GTCCAATCTGGCTAAAGAGTTGC
Human Tial1 (Forw)	Sigma Aldrich	RT-qPCR	AGCATTTGCCCCCTTTGGTA
Human Tial1 (Rev)	Sigma Aldrich		TCGCATTTTCTGCATCCAGTT
Human Hprt (Forw)	Sigma Aldrich	RT-qPCR	TGGACAGGACTGAACGTCTTG
Human Hprt (Rev)	Sigma Aldrich		CCAGCAGGTCAGCAAAGAATTTA
Bicistronic Trp53 IRES vector - Primer 1	Sigma Aldrich	Cloning	AATTCGTTTAAACCTAGAGCTTTCCCCTCCCACGTGCTC
Bicistronic Trp53 IRES vector - Primer 2	Sigma Aldrich	(Gibson assembly)	TGCCAAGCTTCCAGTCTTCGGAGAAGCG
Bicistronic Trp53 IRES vector - Primer 3	Sigma Aldrich		CTCCGAAGACTGGAAGCTTGGCATTCCGGTAC
Bicistronic Trp53 IRES vector - Primer 4	Sigma Aldrich		GGGTAGAAGGGAGCAGGGCCCTTCTTAATGTTCTTAGCATCGG
Bicistronic Trp53 IRES Mutant vector cloning - Primer 1	Sigma Aldrich	Cloning	GGCCTGACATCGAGGAGGATATCGCCCTGATCAAGAGC
Bicistronic Trp53 IRES Mutant vector cloning - Primer 2	Sigma Aldrich	(Gibson assembly)	ACTGTGTCGACGATGGTCCCAATGAACTG
Bicistronic Trp53 IRES Mutant vector cloning - Primer 3	Sigma Aldrich		CTAATTGGGACCATCGTCGACACAGTTAGGGGGCACCTA
Bicistronic Trp53 IRES Mutant vector cloning - Primer 4	Sigma Aldrich		GGGTAGAAGGGAGCAGGGCCCTTCTTAATGTTCTTAGCATCGGC
Trp53 3'UTR (wt) cloning - Forward	Sigma Aldrich	Cloning	AAGAGACTCGAGTGACTGCCTCTGCATCCCGT
Trp53 3'UTR (wt) cloning - Reverse	Sigma Aldrich		AAGAGAGCGGCCGCTAAGTGATAACAAAATTTTATTGTAAAATAGG
Frag1_fwd	Sigma Aldrich	Cloning	TTTGCTCACATGGCTCGACAGATCTGCGCAGCACCATG
Trp53-3'UTR-P_Mut_Frag1_rev	Sigma Aldrich	(Gibson assembly)	CCTAGCTACTAGGTTACATAGGCAGCAGAAGGGACCGG
Trp53-3'UTR-P_Mut_Frag2_forw	Sigma Aldrich		TTCTGCTGCCTATGTAACCTAGTAGCTAGGGCTCAGCC
Trp53-3'UTR_Frag2_rev	Sigma Aldrich		GTAAAAAAAAAAAAAAGAGGGAGACAGGGTGGGG
Trp53-3'UTR-D_Mut_Frag2_rev	Sigma Aldrich		TAACTAGGGGTTACATACATACATAGAGGGAGACAGGGTGGGG
Trp53-3'UTR_Frag3_forw	Sigma Aldrich		CTGTCTCCCTCTTTTTTTTTTTTTTTACCCCTTTTTATATATCAATTTCCTATTTTACAATAAAATTTTGTTATCACTTAG
Trp53-3'UTR-D_Mut_Frag3_forw	Sigma Aldrich		CTGTCTCCCTCTATGTATGTATGTAACCCCTAGTTATATATCAATTTCCTATTTTACAATAAAATTTTGTTATCACTTAG
Frag3_rev	Sigma Aldrich		TCGCCGCCGCGGCGGCGCCGCCCGGGGGTCTGCGAGCAGAC

mTia1 forward primer	Sigma Aldrich	Cloning	AAGAGAAGATCTCGCCACCATGGAGGACGAGATGCCCAAGA
Tia1 ΔRRM1+2 forward primer	Sigma Aldrich		AAGAGAAGATCTCGCCACCATGAAGCCTCCAGCTCCAAAGAGTA
mTia1 reverse primer	Sigma Aldrich		AAGAGAGAATTCGTCTGTTTTCACTGGGTTTCATAC
L34 RNA linker	Sigma Aldrich	iCLIP	P-AUAGAUCGGAAGAGCGGUUCAG-Puromycin
RCLIP Linker F	Sigma Aldrich	iCLIP	5'-P-NNNNGAAAGATCGGAAGAGCGTCGTGGATCCTGAACCGCTC-3’
RCLIP Linker D	Sigma Aldrich	iCLIP	5'-P-NNNNACTAGATCGGAAGAGCGTCGTGGATCCTGAACCGCTC-3’
Cut4c primer	Sigma Aldrich	iCLIP	GTTCAGGATCCACGACGCTCTTCAAAA
Solexa P3 Primer	Sigma Aldrich	iCLIP	CAAGCAGAAGACGGCATACGAGATCGGTCTCGGCATTCCTGCTGAACCGCTCTTCCGATCT
Solexa P5 Primer	Sigma Aldrich	iCLIP	AATGATACGGCGACCACCGAGATCTACACTCTTTCCCTACACGACGCTCTTCCGATCT
2RT primer	IDT	3'end RNAseq	CTGAACCGCTCTTCCGATCTNNNNNNNN
mRNARt_1	IDT	3'end RNAseq	CGAATTTAATACGACTCACTATAGGGAGACACGACGCTCTTCCGATCTAACACNNNNNNCCTTTTTTTTTTTTTTTTTTTTV
mRNARt_2	IDT	3'end RNAseq	CGAATTTAATACGACTCACTATAGGGAGACACGACGCTCTTCCGATCTCACAGNNNNNNCCTTTTTTTTTTTTTTTTTTTTV
mRNARt_3	IDT	3'end RNAseq	CGAATTTAATACGACTCACTATAGGGAGACACGACGCTCTTCCGATCTGAGACNNNNNNCCTTTTTTTTTTTTTTTTTTTTV
mRNARt_4	IDT	3'end RNAseq	CGAATTTAATACGACTCACTATAGGGAGACACGACGCTCTTCCGATCTTACTCNNNNNNCCTTTTTTTTTTTTTTTTTTTTV
mRNARt_5	IDT	3'end RNAseq	CGAATTTAATACGACTCACTATAGGGAGACACGACGCTCTTCCGATCTAAGCANNNNNNCCTTTTTTTTTTTTTTTTTTTTV
mRNARt_6	IDT	3'end RNAseq	CGAATTTAATACGACTCACTATAGGGAGACACGACGCTCTTCCGATCTCAGCCNNNNNNCCTTTTTTTTTTTTTTTTTTTTV
mRNARt_7	IDT	3'end RNAseq	CGAATTTAATACGACTCACTATAGGGAGACACGACGCTCTTCCGATCTGATCANNNNNNCCTTTTTTTTTTTTTTTTTTTTV
mRNARt_8	IDT	3'end RNAseq	CGAATTTAATACGACTCACTATAGGGAGACACGACGCTCTTCCGATCTTATAANNNNNNCCTTTTTTTTTTTTTTTTTTTTV
mRNARt_9	IDT	3'end RNAseq	CGAATTTAATACGACTCACTATAGGGAGACACGACGCTCTTCCGATCTAATCTNNNNNNCCTTTTTTTTTTTTTTTTTTTTV
mRNARt_10	IDT	3'end RNAseq	CGAATTTAATACGACTCACTATAGGGAGACACGACGCTCTTCCGATCTCATGANNNNNNCCTTTTTTTTTTTTTTTTTTTTV
mRNARt_11	IDT	3'end RNAseq	CGAATTTAATACGACTCACTATAGGGAGACACGACGCTCTTCCGATCTGCACTNNNNNNCCTTTTTTTTTTTTTTTTTTTTV
mRNARt_12	IDT	3'end RNAseq	CGAATTTAATACGACTCACTATAGGGAGACACGACGCTCTTCCGATCTTCAATNNNNNNCCTTTTTTTTTTTTTTTTTTTTV
mRNARt_13	IDT	3'end RNAseq	CGAATTTAATACGACTCACTATAGGGAGACACGACGCTCTTCCGATCTACATANNNNNNCCTTTTTTTTTTTTTTTTTTTTV
mRNARt_14	IDT	3'end RNAseq	CGAATTTAATACGACTCACTATAGGGAGACACGACGCTCTTCCGATCTCCAGTNNNNNNCCTTTTTTTTTTTTTTTTTTTTV
mRNARt_15	IDT	3'end RNAseq	CGAATTTAATACGACTCACTATAGGGAGACACGACGCTCTTCCGATCTGCCGANNNNNNCCTTTTTTTTTTTTTTTTTTTTV
mRNARt_16	IDT	3'end RNAseq	CGAATTTAATACGACTCACTATAGGGAGACACGACGCTCTTCCGATCTTCCGGNNNNNNCCTTTTTTTTTTTTTTTTTTTTV
mRNARt_17	IDT	3'end RNAseq	CGAATTTAATACGACTCACTATAGGGAGACACGACGCTCTTCCGATCTACCTTNNNNNNCCTTTTTTTTTTTTTTTTTTTTV
mRNARt_18	IDT	3'end RNAseq	CGAATTTAATACGACTCACTATAGGGAGACACGACGCTCTTCCGATCTCCGTGNNNNNNCCTTTTTTTTTTTTTTTTTTTTV
mRNARt_19	IDT	3'end RNAseq	CGAATTTAATACGACTCACTATAGGGAGACACGACGCTCTTCCGATCTGCGTCNNNNNNCCTTTTTTTTTTTTTTTTTTTTV
mRNARt_20	IDT	3'end RNAseq	CGAATTTAATACGACTCACTATAGGGAGACACGACGCTCTTCCGATCTTCGTCNNNNNNCCTTTTTTTTTTTTTTTTTTTTV
mRNARt_21	IDT	3'end RNAseq	CGAATTTAATACGACTCACTATAGGGAGACACGACGCTCTTCCGATCTACTAGNNNNNNCCTTTTTTTTTTTTTTTTTTTTV
mRNARt_22	IDT	3'end RNAseq	CGAATTTAATACGACTCACTATAGGGAGACACGACGCTCTTCCGATCTCGAACNNNNNNCCTTTTTTTTTTTTTTTTTTTTV
mRNARt_23	IDT	3'end RNAseq	CGAATTTAATACGACTCACTATAGGGAGACACGACGCTCTTCCGATCTGGCAANNNNNNCCTTTTTTTTTTTTTTTTTTTTV
mRNARt_24	IDT	3'end RNAseq	CGAATTTAATACGACTCACTATAGGGAGACACGACGCTCTTCCGATCTTGACANNNNNNCCTTTTTTTTTTTTTTTTTTTTV
mRNARt_25	IDT	3'end RNAseq	CGAATTTAATACGACTCACTATAGGGAGACACGACGCTCTTCCGATCTAGACCNNNNNNCCTTTTTTTTTTTTTTTTTTTTV
mRNARt_26	IDT	3'end RNAseq	CGAATTTAATACGACTCACTATAGGGAGACACGACGCTCTTCCGATCTCGCCANNNNNNCCTTTTTTTTTTTTTTTTTTTTV
mRNARt_27	IDT	3'end RNAseq	CGAATTTAATACGACTCACTATAGGGAGACACGACGCTCTTCCGATCTGGCATNNNNNNCCTTTTTTTTTTTTTTTTTTTTV
mRNARt_28	IDT	3'end RNAseq	CGAATTTAATACGACTCACTATAGGGAGACACGACGCTCTTCCGATCTTGCATNNNNNNCCTTTTTTTTTTTTTTTTTTTTV
mRNARt_29	IDT	3'end RNAseq	CGAATTTAATACGACTCACTATAGGGAGACACGACGCTCTTCCGATCTAGCGANNNNNNCCTTTTTTTTTTTTTTTTTTTTV
mRNARt_30	IDT	3'end RNAseq	CGAATTTAATACGACTCACTATAGGGAGACACGACGCTCTTCCGATCTCGGCTNNNNNNCCTTTTTTTTTTTTTTTTTTTTV
mRNARt_31	IDT	3'end RNAseq	CGAATTTAATACGACTCACTATAGGGAGACACGACGCTCTTCCGATCTGGTCGNNNNNNCCTTTTTTTTTTTTTTTTTTTTV
mRNARt_32	IDT	3'end RNAseq	CGAATTTAATACGACTCACTATAGGGAGACACGACGCTCTTCCGATCTTGGCGNNNNNNCCTTTTTTTTTTTTTTTTTTTTV
mRNARt_33	IDT	3'end RNAseq	CGAATTTAATACGACTCACTATAGGGAGACACGACGCTCTTCCGATCTAGGATNNNNNNCCTTTTTTTTTTTTTTTTTTTTV
mRNARt_34	IDT	3'end RNAseq	CGAATTTAATACGACTCACTATAGGGAGACACGACGCTCTTCCGATCTCGTGGNNNNNNCCTTTTTTTTTTTTTTTTTTTTV
mRNARt_35	IDT	3'end RNAseq	CGAATTTAATACGACTCACTATAGGGAGACACGACGCTCTTCCGATCTGTAGCNNNNNNCCTTTTTTTTTTTTTTTTTTTTV
mRNARt_36	IDT	3'end RNAseq	CGAATTTAATACGACTCACTATAGGGAGACACGACGCTCTTCCGATCTTGTTCNNNNNNCCTTTTTTTTTTTTTTTTTTTTV
mRNARt_37	IDT	3'end RNAseq	CGAATTTAATACGACTCACTATAGGGAGACACGACGCTCTTCCGATCTAGTTGNNNNNNCCTTTTTTTTTTTTTTTTTTTTV
mRNARt_38	IDT	3'end RNAseq	CGAATTTAATACGACTCACTATAGGGAGACACGACGCTCTTCCGATCTCTATCNNNNNNCCTTTTTTTTTTTTTTTTTTTTV
mRNARt_39	IDT	3'end RNAseq	CGAATTTAATACGACTCACTATAGGGAGACACGACGCTCTTCCGATCTGTCTANNNNNNCCTTTTTTTTTTTTTTTTTTTTV
mRNARt_40	IDT	3'end RNAseq	CGAATTTAATACGACTCACTATAGGGAGACACGACGCTCTTCCGATCTTTCAGNNNNNNCCTTTTTTTTTTTTTTTTTTTTV
mRNARt_41	IDT	3'end RNAseq	CGAATTTAATACGACTCACTATAGGGAGACACGACGCTCTTCCGATCTATCACNNNNNNCCTTTTTTTTTTTTTTTTTTTTV
mRNARt_42	IDT	3'end RNAseq	CGAATTTAATACGACTCACTATAGGGAGACACGACGCTCTTCCGATCTCTGAANNNNNNCCTTTTTTTTTTTTTTTTTTTTV
mRNARt_43	IDT	3'end RNAseq	CGAATTTAATACGACTCACTATAGGGAGACACGACGCTCTTCCGATCTGTGTTNNNNNNCCTTTTTTTTTTTTTTTTTTTTV
mRNARt_44	IDT	3'end RNAseq	CGAATTTAATACGACTCACTATAGGGAGACACGACGCTCTTCCGATCTTTGCCNNNNNNCCTTTTTTTTTTTTTTTTTTTTV

mRNARt_45	IDT	3'end RNAseq	CGAATTTAATACGACTCACTATAGGGAGACACGACGCTCTTCCGATCTATGCANNNNNNCCTTTTTTTTTTTTTTTTTTTTV
mRNARt_46	IDT	3'end RNAseq	CGAATTTAATACGACTCACTATAGGGAGACACGACGCTCTTCCGATCTCTTATNNNNNNCCTTTTTTTTTTTTTTTTTTTTV
mRNARt_47	IDT	3'end RNAseq	CGAATTTAATACGACTCACTATAGGGAGACACGACGCTCTTCCGATCTTAAGGNNNNNNCCTTTTTTTTTTTTTTTTTTTTV
mRNARt_48	IDT	3'end RNAseq	CGAATTTAATACGACTCACTATAGGGAGACACGACGCTCTTCCGATCTTTGTANNNNNNCCTTTTTTTTTTTTTTTTTTTTV
mRNARt_49	IDT	3'end RNAseq	CGAATTTAATACGACTCACTATAGGGAGACACGACGCTCTTCCGATCTATTCTNNNNNNCCTTTTTTTTTTTTTTTTTTTTV
mRNARt_50	IDT	3'end RNAseq	CGAATTTAATACGACTCACTATAGGGAGACACGACGCTCTTCCGATCTGAATGNNNNNNCCTTTTTTTTTTTTTTTTTTTTV
